# Genetic Polymorphism Analysis of 24 Y-STRs in a Han Chinese Population in Luzhou, Southwest China

**DOI:** 10.3390/genes14101904

**Published:** 2023-10-02

**Authors:** Jiewen Fu, Binghui Song, Jie Qian, Ting He, Hanchun Chen, Jingliang Cheng, Junjiang Fu

**Affiliations:** 1 Key Laboratory of Epigenetics and Oncology, The Research Center for Preclinical Medicine, Southwest Medical University, Luzhou 646000, China; fujiewen@swmu.edu.cn (J.F.); songbinghui@stu.swmu.edu.cn (B.S.); 20210199120039@stu.swmu.edu.cn (J.Q.); 20220199120039@stu.swmu.edu.cn (T.H.); jingliangc@swmu.edu.cn (J.C.); 2 School for Basic Medicine, Southwest Medical University, Luzhou 646000, China; 3 Laboratory of Forensic DNA, The Judicial Authentication Center, Southwest Medical University, Luzhou 646000, China; 4 Department of Biochemistry and Molecular Biology, School of Life Sciences, Central South University, Changsha 410013, China; chenhanchun@csu.edu.cn

**Keywords:** Y-STRs, forensic genetics, genetic polymorphism, Han Chinese population

## Abstract

Han is the largest of China’s 56 ethnic groups and the most populous ethnic group in the world. The Luzhou region is located in southwest China, at the junction of three provinces. The unique historical factors contribute to the genetic polymorphism information. Short tandem repeats (STRs) are highly polymorphic, but the polymorphism of the Y chromosomal STRs (Y-STRs) loci in the Luzhou region is still unclear. It is of great significance to provide Y-STRs genetic data for the Han population from the Luzhou areas of southwest China. A total of 910 unrelated male individuals of the Han population from the Luzhou area were recruited, and 24 Y-STRs were analyzed. The population structure and phylogenetic relationships were compared with those of another 11 related Han populations. A total of 893 different haplotypes were achieved from 910 samples, of which 877 (98.21%) haplotypes were unique. Haplotype diversity and discrimination were 0.999956 and 0.981319, respectively. The lowest genetic diversity of DYS437 is 0.4321, and the highest genetic diversity of DYS385a/b is 0.9642. Pair-to-pair genetic distance and relative probability values indicate that Luzhou Han people are close to Sichuan Han people, Guangdong Han people, and Hunan Han people, which is consistent with geographical distribution, historical influence, and economic development. The 24 Y-STR markers of the southwest Luzhou Han population were highly polymorphic, which provided us with genetic polymorphism information and enriched the population genetic database. Therefore, it is of great value to our forensic applications and population genetics research.

## 1. Introduction

Short tandem repeats (STRs) are highly polymorphic and widely used in forensic science, anthropology, and genetics. STR genetic markers can be categorized into autosomal short tandem repeats (A-STRs), Y chromosome short tandem repeats (Y-STRs), and X chromosome short tandem repeats (X-STRs), according to their distribution location on chromosomes. The unique genetic approach of sex chromosomes makes them extremely advantageous in the identification of victims of mass disaster events, the investigation of family lineages needed for missing person cases, and the testing of mixed samples in sexual assault crimes. Y-STRs have the characteristics of paternal inheritance, and the results of Y-STR typing of male individuals in the same family are consistent. Y-STR polymorphism analysis is of great forensic practical value, which plays a critical role in the investigation and solving of cases for forensic casework [1,2]. Their usability and purpose for human identification are limited but can be helpful to enhance the evidence and completely differentiate, for example, in sexual assault cases [1]. Moreover, because Y-STRs are paternally inherited by male offspring and the Y-STR haplotypes within the same paternal lineage are consistent, they have great application value in the study of human origin, evolution, migration, and clan relationships [3]. Through the construction of the Y-STR library, the power of DNA technology can be more effectively exerted. It would be a useful addition to the A-STR repertoire. For example, a violent murder occurred in 2009. Through increasing the number of detected Y-STRs and the combined calculation of multiple A-STRs and the kinship index, Yang et al. [4] successfully identified the perpetrator as a previously unknown illegitimate child of a large family and solved the case in 2021. Their usability and purpose are helpful in enhancing the evidence for sexual assault cases [5]. In particular, they play a role in the personal identification of mixed stains and paternity identification in paternity testing of gang rape cases. Therefore, Y-STR markers have an important application value in forensic genetics. 

Y-STR haplotypes are a combination of Y-STR loci alleles. It is necessary to combine multiple Y-STR loci to obtain more haplotypes for improving individual identification ability. At present, there are commonly used commercial Y-STR kits containing multiple markers including PowerPlex^®^ Y kits, PowerPlex^®^ Y23 kits, Yfiler kits, Yfiler TM Plus kits, etc. [6,7]. The PowerPlex^®^ Y23 Kit has been widely used in the analysis of population data and the construction of Y-STR databases, including different ethnic populations in many countries, such as China [8], The United Kingdom [9], Brazil [10], Serbia [11], and so on. The Microreader™24Y Direct ID System includes all 23 Y-STR markers of the PowerPlex Y23 system and another DYS460 locus, i.e., 24 Y-STR loci in total. Therefore, markers in this kit are highly polymorphic [12]. 

Y-STRs were assigned names by the gene nomenclature committee (HGNC), a committee of the Human Genome Organization (HUGO) that sets the standards for the nomenclature of human genes including STRs. Y-STRs are taken specifically from the Y chromosome in males, which may provide a weaker analysis than A-STRs. The traditional Y-STR system can be used to identify male pedigrees, but it is more difficult to use it to distinguish related male individuals. Thus, the rapidly mutating Y-chromosomal STRs (RM Y-STRs) have relatively high mutation rates (>1 × 10^2^ mutation rates than common Y-STRs) and may play an important role in identifying related males. Until now, a large number of studies have been conducted on RM Y-STRs [13,14], which can provide a powerful tool for Y chromosome analysis in forensic genetics, including migratory events in the Luzhou area that would have affected the Y-STR haplotype diversity. In a previous study, our group investigated the 23 Y-STR mutation rates of this system in the southwest Han population [15]. In 175 father-son sample pairs, 16 repeat mutations at 10 loci including three mutations at DYS570, two mutations at DYS549, DYS458, DYS460, and DYS576, and one mutation at another five Y-STRs were found. All the observed repeat mutations showed single repeat changes such as repeat insertions or repeat deletions. Thus, RM Y-STRs are important for kinship analysis, paternal lineage identification, and family relationship reconstruction.

**Figure 1:**
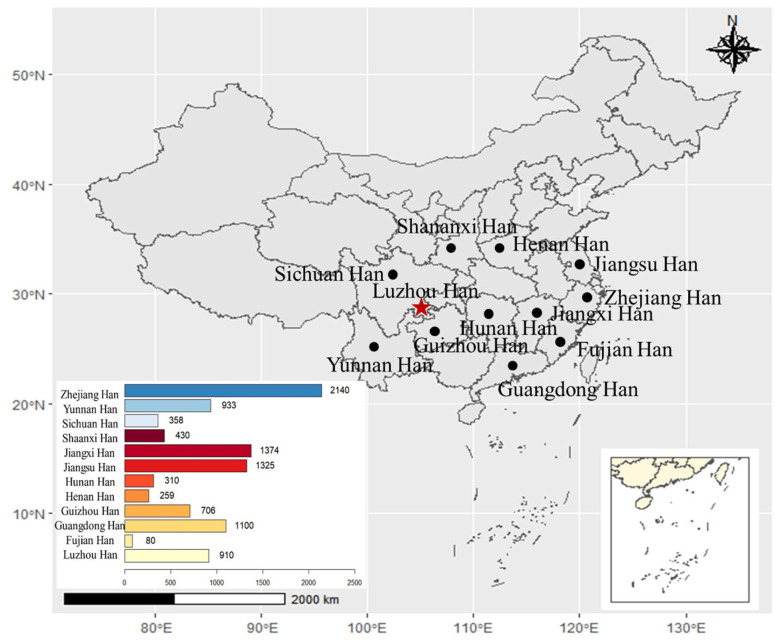
Map showing the approximate geographic positions of the studied Han population in Luzhou areas of southwest and other 11 reference populations in China. The number of haplotypes (the number of samples) in different populations is presented at the bottom left of the figure. The R project software (version 4.0.5) (https://www.r-project.org/) (accessed on 29 December 2021) was used to build this map. The star in red represents Luzhou City. The circle “•” in black represents the other 11 areas.

Luzhou, a city in southwest China, is situated upstream of the Yangtze River in southeast Sichuan Province and is at the border between Sichuan Province, Guizhou Province, Yunnan Province, and Chongqing City (please see Figure 1). Yangtze River, or “Long River”, is the longest in the world to flow entirely within one country and the third-longest river in the world. The geographical coordinates in Luzhou are between 27°39′ to 29°20′ north latitude and 105°8′ to 106°28′ east longitude. Based on China’s Seventh National Census Bulletin, the permanent population of Luzhou is 4,254,149, which was published online on 27 May 2021 (http://tjj.luzhou.gov.cn/tjsj/tjgb/content_802318) (accessed on 29 December 2021). Compared with China’s Sixth National Census ten years ago, the increase in the population is 35,723, its growth rate is 0.85% and the average annual growth rate is 0.08%. Although all ethnic groups are rich in population resources, and the Han nationality is the main group in this region, its relevant population structure and genetic characteristics have not been deeply studied. Moreover, Luzhou has gone through several population migrations since the Qin Dynasty, the most famous being the “Huguang Tian Sichuan” Immigration Movement in the Qing Dynasty. The Han population has integrated with different ethnic groups here, forming a unique and complex group relationship in the region. Thus, it is interesting and important to analyze Y-STRs in the Han population in the Luzhou area and the historical factors that contributed to male lineage diversity. However, studies on the genetic polymorphism of Y-STR in Luzhou, southwest China are scarce, and the number of Y-STR markers and the number of samples are not enough [16]. Herein, we used 24 Y-STR loci (Microreader™ 24Y Direct ID System) to explore genetic polymorphism in the Han Chinese population from the Luzhou areas, and the suspected STR genotyping was verified using the Microreader™ 29Y Direct ID System or the Yfiler™ Plus PCR Amplification Kit.

## 2. Materials and Methods

### 2.1. Regents

The Microreader™ 24Y Direct ID System was purchased at Microread Genetics Co., Ltd., Suzhou, China, including 24 Y-STR loci, namely DYS19, DYS385ab, DYS391, DYS392, DYS393, DYS570, DYS549, DYS448, DYS458, DYS481, DYS460, DYS635, DYS533, DYS456, DYS389I, DYS390, DYS389II, DYS438, DYS576, DYS439, DYS437, DYS643, and Y GATA H4. The Microreader™ 29Y Direct ID System (Cat. #: 10401207, Microread Genetics Co., Ltd., Suzhou, China) or the Yfiler™ Plus PCR Amplification Kit (Cat. #: 4482730, Thermo Fisher Scientific, Waltham, MA, USA) was also applied to verify the suspected STR genotyping. The Yfiler™ Plus PCR Amplification Kit covers 27 Y-STRs including 7 rapidly mutating (RM) Y-STRs with >1 × 10^2^ mutation rates, while the Microreader™ 29Y Direct ID System covers 29 Y-STRs including 7 RM Y-STRs. FTA cards were purchased from Shandong Chengwu Ronghua Biotechnology Co., Ltd., Heze, China (Cat. #: 20201127). Chelex-100 was purchased at Bio-Rad Laboratories Co., Ltd., Hercules, CA, USA (Cat. #: 143-2832). Proteinase K and phenol/chloroform were purchased from Merck KGaA, Darmstadt, Germany (Cat. #: 1245680100) and Beijing Solarbio Science & Technology Co., Ltd., Beijing, China (Cat. #: T0250), respectively.

### 2.2. Sample Recruitment and Genomic DNA Extraction

Under informed consent, we recruited blood samples from 910 unrelated male individuals of the Han population in the Luzhou areas, including Sichuan Province, Guizhou Province, Yunnan Province, and Chongqing City in southwest China (Figure 1). The Chelex-100 method [17,18,19] was used to extract genomic DNA (gDNA) from FTA cards, and the protease K/phenol/chloroform method was used to extract gDNA from EDTA-coated blood samples [20,21,22]. For details regarding the chelex-100 method: a hole punch was used to collect two 1 mm diameter blood spot samples from the FTA card. It was then placed in a 600 µL centrifuge tube. A total of 400 µL double distilled water (ddH_2_O) was supplied into a 600 µL Eppendorf (EP) tube, mixed upside down for 2–3 s, and left at room temperature for 15 min. After centrifuging at 13,200× *g* for 1 min, the blood spot sample was submerged at the bottom of the EP tube. Then the supernatant was removed, 35 µL 5% chelex-100 was supplied, and the sample was bathed at 56 °C for 30 min. Then the samples were boiled directly in boiling water for 8 min, and finally, the centrifuge tube was turned into a high-speed centrifuge (Thermo Fisher, Waltham, MA, USA) at 13,200× *g* for 1 min. The 20 µL supernatant was transferred to a new 600 µL EP tube, which can then be used directly for polymerase chain reaction (PCR) amplification. Samples were stored in the refrigerator at −20 °C for future use. 

For whole blood samples, we used the protease K/phenol/chloroform method to extract genomic DNA. DNA extraction from whole blood samples was performed because we have a DNA library for normal samples which can store larger amounts of DNA of better quality for further use. For details, we first took 2 mL of whole blood and placed it in a 10 mL EP tube, added 2.5 times the volume of 1 × red blood cell lysate (1.55 mol/L NH_4_Cl, 0.1 mol/L KHCO_3_, 0.001 mol/L EDTA (pH 8.0)), mixed by inversion to make it fully lysate, and then placed it into an ice bath for 15 min before centrifuging for 10 min in a low-speed centrifuge (825× *g*). The supernatant was removed, 700 µL of 1 × leukocyte lysate (0.001 mol/L Tris, 0.04 mol/L Nacl, 0.0002 mol/L EDTA (pH 8.0)) was added, mixed well, and then transferred to a new 1.5 ml EP tube with 80 µL of 20% SDS and 7 µL of protease K (20 mg/mL). Next, it was mixed and bathed in water at 56 °C for 4 h. Then, an equal volume of balanced phenol was added, and it was shaken and mixed. Next, an equal volume of balanced phenol was added, shaken, and mixed again, before being high-speed centrifuged at 13,200× *g* for 5 min. Approximately 400 µL of supernatant was transferred to a new 1.5 ml EP tube, and an equal volume of phenol/chloroform was supplied. After shaking and mixing, high-speed centrifugation was performed at 13,200× *g* for 5 min. Next, we transferred 250 µL of supernatant to a new 1.5 mL EP tube, added 4 times the volume of anhydrous ethanol, mixed upside down, and centrifuged at 13,200× *g* for 1 min. The supernatant was removed, washed twice with 75% ethanol, then sterilized water was added to dissolve the DNA, which can be directly used for polymerase chain reaction (PCR) amplification. Samples were also stored in the refrigerator at −20 °C for future use. 

### 2.3. PCR Amplification and STR Genotyping 

The 24 Y chromosome STR loci were amplified using the Microreader 24Y^TM^ Direct ID system (Shandong Chengwu Ronghua Biotechnology Co., Ltd., China, Lot: 20210115) according to the manufacturer’s instructions. The Y-STR loci were amplified in an Applied Biosystems Veriti^®^ 96-Well Thermal Cycler (Applied Biosystems, Life Technology, USA). For details, each sample used 10 μL PCR reaction volume, including DNA sample 1 µL, ddH_2_O 2.8 µL, Microreader™ 24Y-D 5 × Primer Mix 2 µL, Microreader™ Taq DNA Polymerase II 0.2 µL, and Microreader™ 2.5 × Buffer B 4 µL. The steps of PCR amplification were performed as previously described [22,23]. For details, The PCR reaction was set to the following program: pre-denaturation at 96 °C for 2 min; denaturation at 94 °C for 5 s, annealing and extension at 60 °C for 70 s, cycle number was 28, and final extension at 60 °C for 30 min, maintenance at 16 °C. 

After PCR amplification, the 10 µL mixture system was genotyped by capillary electrophoresis, including 1 µL PCR products, deionized formamide 8.5 μL, and Microreader™ Size Standard Org 500 0.5 μL. For quality, control DNA M308 was also added to this kit. Blank control (no DNA template) was used as a negative control to ensure that there was no contamination during the experiment. Genotyping and analysis were performed using the ladder that came with the kit. Our center gets the certification from the China National Accreditation Service for Conformity Assessment (CNAS) and Calibration Laboratories Accreditation Criteria for the Competence of Testing and Calibration Laboratories. Genotype analysis for Y-STRs was conducted by referencing the Specification of Y-STR Testing for Forensic Purpose by China (SF/Z JD0105007-2018) and the International Society of Forensic Genetics’ (ISFG) recommendations for analyzing the Y-STR markers [24]. Then the amplified PCR products were used to genotype by capillary electrophoresis on the Genetic Analyzer (3500 DX, Applied Biosystems, Life Technology, USA), and the results were analyzed by the GeneMapper ID-X software (Thermo Fisher, Waltham, MA, USA) [23]. For details, open the analysis software “GeneMapper-X 1.5” and click the “Add sample to project” button to import the electrophoresis file to be analyzed. After import, select “MR24Y_STR_ID” in “Analysis method”, “MR24Y Kits V1.3” in “Panel”, and “Org 500” in “Size Standard”. The STR typing of each sample can be obtained by clicking the “Analysis” button after selecting “Positive Control”, “Negative Control”, and “Allelic Ladder” for M308, Negative, and Ladder samples, respectively.

### 2.4. Data Analysis

The indices to evaluate the differential ability of a Y-STR typing system are haplotype diversity (HD) and genetic diversity (GD), calculated by a direct counting method using the formulas HD = N(1 − ∑pi^2^)/(N − 1) and GD = N(1 − ∑ai^2^)/(N − 1), where *N* is the total number of the target population, *pi* is the frequency of the *i*th haplotype, and ai is the frequency of the *i*th allele. The discrimination capacity (DC) was calculated as DC = M/N, where *M* is the number of different haplotypes, and *N* is the total number of the target population. The haplotype match probability (HMP) was measured using the formula HMP = ∑pi^2^, where *pi* is the frequency for the *i*th haplotype. We used the online tools for the Y chromosome haplotype reference database (YHRD) [25,26] (https://www.yhrd.org/) (accessed on 29 December 2021) to obtain the pairwise genetic distances (Rst) through Analysis of Molecular Variance (AMOVA), and the multidimensional scaling (MDS) plots based on the Rst values to reveal the population relationships. For details, firstly we selected “AMOVA&MDS” for the Y-STR data analysis under the “Tools” option of the website, and secondly, based on the haplotype data of Luzhou Han and databases of relevant Han Chinese populations in the YHRD, we calculated the Rst and built the MDS plot. Through this method, we can download the analysis results from this website and obtain references of relevant Han Chinese populations. Then the heatmap of Rst between these populations was plotted by OriginPro 2023b software (version 10.0.5.153) [27]. In addition, MEGA-X software (version 10.0.5) [28] was used to construct the neighbor-joining phylogenetic tree based on the Rst values.

## 3. Results

### 3.1. Genetic Polymorphisms and Forensic Characteristics

The haplotype data for 24 Y-STRs in the Luzhou Han population are listed in Supplementary Table S1. A total of 893 different haplotypes were obtained from 910 samples, among which 877 (98.21%) haplotypes were unique, 15 (1.68%) haplotypes occurred twice, and one (0.11%) haplotype occurred thrice. There was a total of three microvariants at one Y-STR locus: DYS458. It had three different microvariants, including 13.1, 14.1, and 15.1. Furthermore, a total of five samples were observed copy-number variants. DYS385a/b had a tri-allelic genotype, three tetra-allelic genotypes, and a pentagenic genotype. The microvariants of locus DYS458 and the copy-number variants of locus DYS385a/b have been frequently observed in previous research on the Han or global population [15,23,29,30]. As shown in Supplementary Figure S1, the eight samples were also detected using the Yfiler™ Plus PCR Amplification Kit (Supplementary Figure S1B) or Microreader™ 29Y Direct ID System (Supplementary Figure S1C), and the result is consistent with the genotyping obtained using the Microreader™24Y Direct ID System (Supplementary Figure S1A).

Then, the allele frequencies and GD values of 24 Y-STR loci including 22 single-copy and one multi-copy Y-STR loci are shown in Supplementary Table S2. Among 22 single-copy Y-STR loci, the number of alleles varied from four in locus DYS437 to 17 in locus DYS481, and allele frequencies varied from 0.0011 to 0.7033. The lowest GD value was 0.4321 in locus DYS437 and the highest was 0.8274 in locus DYS481, followed by 0.8198 in locus DYS458. A multi-copy Y-STR locus DYS385a/b owned more genetic information than all of the 22 single-copy Y-STR loci, with a GD value of 0.9642. Furthermore, twenty of 24 Y-STR loci exhibited GD values greater than 0.6, which demonstrated that the Luzhou Han population had high genetic diversity. 

We further evaluated the forensic parameters in four YHRD separate datasets (Minimal, PowerPlex Y12, Yfiler, and PowerPlex Y23) and the Microreader 24Y System, and the results are shown in Table 1. It is found that the increasing number of Y-STR markers improved HD and DC values and decreased HMP values. In the nine minimal loci systems, the discrimination capacity was only 0.7703, including 701 different haplotypes. After the addition of 15 Y-STR markers, the DC values were significantly increased to 0.9813 in the Microreader 24Y System. The number of different haplotypes increased by 27.39% to 893. In addition, the Microreader 24Y System produced the highest HD value of 0.999956.

### 3.2. Population Structure and Population Genetic Relationship 

**Figure 2:**
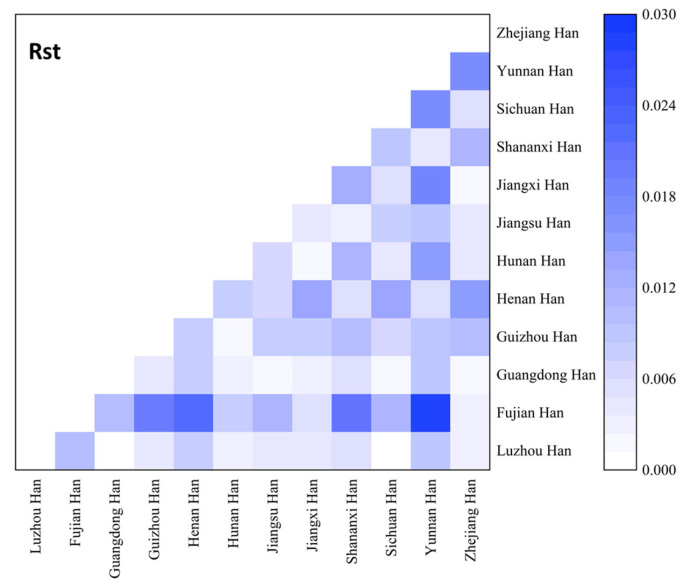
Heatmap of the pairwise genetic distances (Rst) of the Luzhou Han population and the other 11 reference populations.

**Figure 3:**
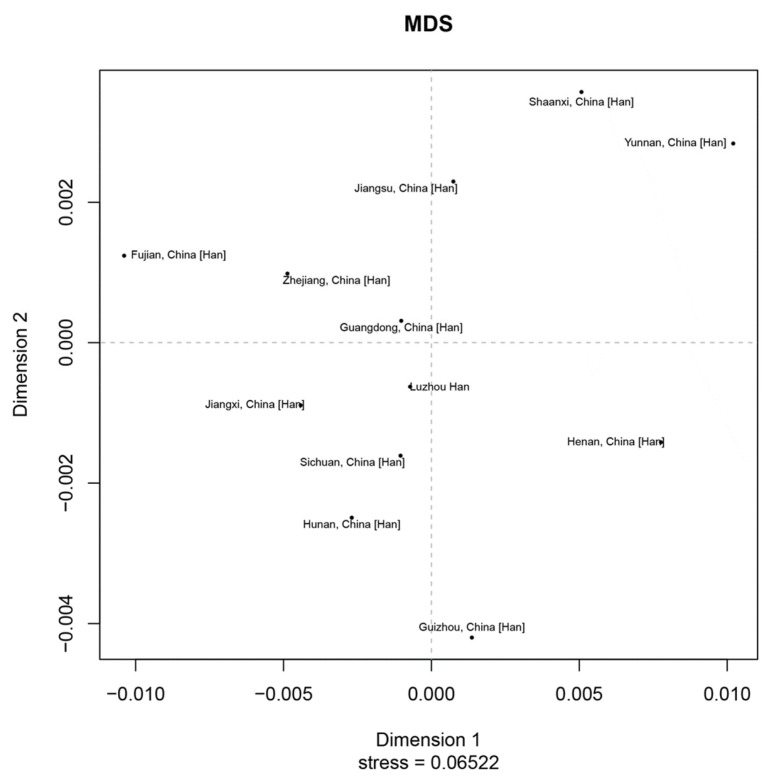
Multidimensional Scaling plots displayed the genetic relationships based on Rst values between the Luzhou Han and the other 11 reference populations.

**Figure 4:**
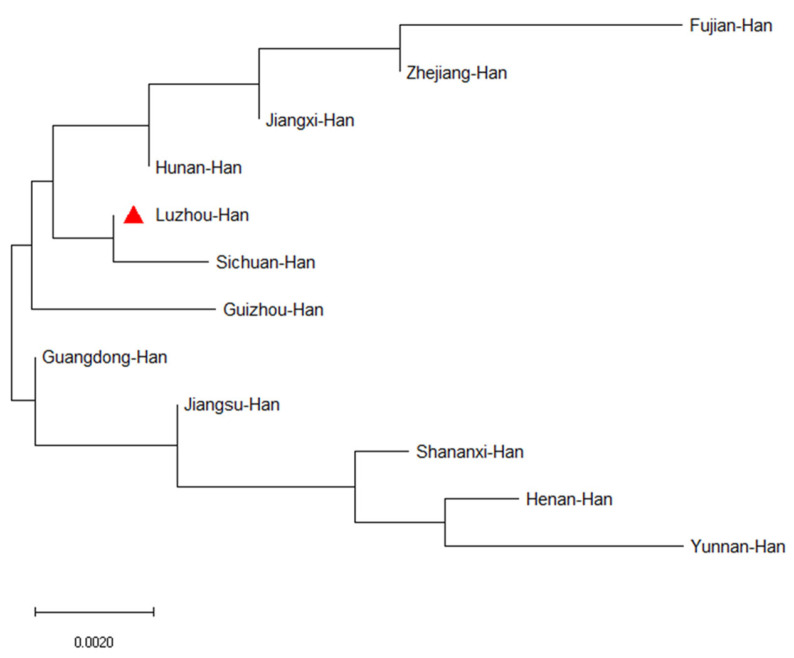
The phylogenetic tree displayed the genetic relationships between the Luzhou population and the other 11 reference populations. The phylogenetic tree was constructed using the neighbor-joining method based on 23 Y-STR loci with MEGA-X software. The red triangle indicates the work in this study.

In order to study population structure and phylogenetic relationships between Luzhou Han and relevant Han Chinese populations, we selected previously reported population data in YHRD. As a result, a total of 11 Han populations were chosen as comparison populations, including Henan Han [31], Fujian Han [32], Jiangsu Han [33], Guangdong Han [34], Guizhou Han [35], Hunan Han [36], Jiangxi Han [32], Shananxi Han [37], Sichuan Han [38], Yunnan Han [39], and Zhejiang Han [40], and Figure 1 depicts the approximate geographical location of the Han population in 17 different regions in China. The pairwise Rst values and associated *p*-values between the Luzhou Han population and the other 11 Han populations are shown in Supplementary Table S3. We didn’t find significant differences in genetic distances between Luzhou Han and Fujian Han, Guangdong Han, Guizhou Han, Hunan Han, and Sichuan Han, according to *p*-values after Bonferroni correction (*p* > 0.00076). The results demonstrated that the Luzhou Han had the nearest genetic distance with Sichuan Han (Rst = 0.0008), Guangdong Han (Rst = 0.0008), and Hunan Han (Rst = 0.0026). The heatmap of Rst is shown in Figure 2. From Figure 2, we can observe that the Rst between these populations is more detailed. From Figure 3, Luzhou Han, Sichuan Han, Hunan Han, Guangdong Han, Jiangxi Han, and Zhejiang Han were distributed in the middle part of the MDS plot and were far apart from other Han populations. Moreover, in the neighbor-joining phylogenetic tree (Figure 4), Luzhou Han and Sichuan Han clustered at the same branch. Notably, Luzhou Han was clustered closely with Hunan Han and Guizhou Han. These results were closely associated with the geographical distribution of Han populations, suggesting the influence of social environments and geographical features.

## 4. Discussion

China currently has a population of 1,443,497,378. It is the world’s most populous country, among which the Han population numbers 12,86311,334, accounting for 91.11%. Compared with China’s Sixth National Census, the increase in the Han population was 60,378,693, with a growth rate of 4.93% (http://www.stats.gov.cn/zt_18555/zdtjgz/zgrkpc/dqcrkpc/ggl/202302/t20230215_1903998.html, accessed on 10 September 2023). Han nationality is the most populous nation in the world, and it has different living customs and cultural characteristics in different regions of China. Located in the southwest of China, Luzhou is the junction of Sichuan Province, Yunnan Province, Guizhou Province, and Chongqing City. In these areas, there are many ethnic minorities, which together with the Han nationality form the unique group structure and genetic characteristics of this region. Due to the high mutation rate of Y-STR loci, it can reflect population structure, population differentiation, and phylogenetic relationships of different populations in a relatively short time. However, the Y-STR data of the Han population in Luzhou is scarce and dated.

In the current study, a total of 893 different haplotypes were obtained from 910 samples, of which 877 (98.21%) haplotypes were unique. The same haplotypes might reflect that these individuals came from the same patrilineal inheritance or shared ancestry. Increasing the number of Y-STR loci, these individuals might also be distinguished. Moreover, the haplotype diversity and discrimination capacity were 0.999956 and 0.981319, respectively. The lowest genetic diversity value was 0.4321 in locus DYS437 and the highest was 0.9642 in locus DYS385a/b. Of course, it is no surprise that increasing the number of Y-STR markers improved HD and DC values and decreased HMP values. It is proven that the 24 Y-STR markers are polymorphic and discriminative in the Luzhou Han population and are appropriate for mass disaster events, identifying paternal lineages, and detecting crime samples.

Population structure and phylogenetic relationships between the Luzhou Han Chinese group and the other 11 relevant Han Chinese groups from the Y chromosome haplotype reference database were compared. The pairwise Rst values and associated *p*-values of AMOVA between the Luzhou Han group and the other 11 Han groups revealed that the Luzhou Han are close with Sichuan Han, Guangdong Han, and Hunan Han populations in genetic relationships, but distantly related to the Han population of Fujian. The MDS plot showed that Luzhou Han, Sichuan Han, and Guangdong Han are close to each other, but the Han populations in Guizhou province, Shaanxi, and Yunnan are far away. In addition, the neighbor-joining phylogenetic tree showed that Luzhou Han was clustered closely with Sichuan Han, Hunan Han, and Guizhou Han. Among these Han populations, the Fujian Han and Yunnan Han have more distant genetic relationships with Luzhou Han. Although belonging to the same ethnic group, the Han population showed different genetic distances in various places. These also demonstrated that Han Chinese groups from different geographical regions have genetic differences. Distinct cultures, geographic isolation, and differences in intermarriage may have developed in different geographic areas as a result of geographical distance. The closer the geographical distance, the more homogeneous it tends to be, while the farther it is, the more different it is, which explains the diversity of genetic polymorphic information in the Han population. These results proved that geographical distribution, historical influences, economic development, and cultural integration exerted an influence on the population structure and phylogenetic relationships.

In our previous study, we were the first to examine allele frequencies and forensic parameters of 21 A-STR markers in Luzhou Han and the population genetic structure between this Luzhou population and other Chinese populations [19]. In addition, 10 Y-STR loci, including DYS570, DYS549, DYS460, DYS458, DYS576, Y-GATA-H4, DYS635, DYS389II, DYS438, and DYS385ab, were discovered to possess mutations in 175 Chinese father-son pairs [15]. In this study, we first explored allele frequencies and forensic parameters of 24 Y-STR markers in Luzhou Han and the population genetic structure between this studied population and other Chinese populations. The results are similar to previous studies in that the Luzhou Han are significantly closely associated with the Sichuan Han, Guangdong Han, and Hunan Han genetically. The results are also consistent with historical events, reflecting the impact of population migration on genetic diversity. Combining both markers could provide more useful information for population genetics and practice in forensic medicine in Luzhou. 

## 5. Conclusions

In conclusion, our study reveals that the Luzhou Han population is highly genetically polymorphic, including in the allele frequencies, GD values, and forensic characteristic data, in the 24 Y-STR loci, thus could provide useful information for population genetics and practice in forensic medicine in these areas. The research might not only enrich the Y-STR research of population structure, population genetic relationships, and population genetic differentiation in the Luzhou areas of southwest China but also reveal diversity and admixture in these Han populations. This study also ensures the scientific accuracy of the identification results and provides a useful tool for judicial expertise in southwest China.

## Figures and Tables

**Table 1:** The forensic parameters were evaluated in the five datasets of Y-STR loci in the Han population from the Luzhou area (n = 910).

Count of Observed Haplotype	Minimal	PowerPlex Y	Yfiler	PowerPlex Y23	Microreader 24Y
1	590	674	813	867	877
2	74	70	40	20	15
3	16	12	2	1	1
4	9	6	1	-	-
5	3	3	-	-	-
6	5	1	-	-	-
7	-	1	1	-	-
8	-	1	-	-	-
9	2	-	-	-	-
10	1	-	-	-	-
11	-	-	-	-	-
12	-	-	-	-	-
13	-	-	-	-	-
14	-	-	-	-	-
15	1	-	-	-	-
Number of different haplotypes	701	768	857	888	893
Haplotype match probability	2.31 × 10^3^	1.67 × 10^3^	1.28 × 10^3^	1.15 × 10^3^	1.14 × 10^3^
Haplotype diversity	0.998784	0.999429	0.999823	0.999944	0.999956
Discrimination capacity	0.770330	0.843956	0.941758	0.975824	0.981319

## Data Availability

All data used for the analyses in this report are available from the corresponding author on reasonable request.

## Supplementary Materials

The following supporting information can be downloaded at: https://www.mdpi.com/article/10.3390/genes14101904/s1, Figure S1: A. Electropherograms showing the copy-number variants of locus DYS385a/b in five samples by Microreader™24Y Direct ID System. B. The copy-number variants of locus DYS385a/b in five samples were checked by the Yfiler™ Plus PCR Amplification Kit. C. The microvariants of locus DYS458 in three samples were checked by the Microreader™ 29Y Direct ID System; Table S1: 24 Y-STR haplotypes of Han population in Luzhou, China (n = 910); Table S2: Allele frequencies and genetic diversities (GD) of 24 Y-STR loci in Han population from Luzhou, China; Table S3: The pairwise genetic distances (Rst) and associated probability values (*p*-values) between the Luzhou Han population and the other 11 populations.
