# Supplementary material for: Genetic Polymorphism Analysis of 24 Y-STRs in a Han Chinese Population in Luzhou, Southwest China
Source: Genes (Basel). 2023 Oct 2;14(10):1904. doi: 10.3390/genes14101904 (PMC10606688; doi:10.3390/genes14101904)
Supplement: Supplementary file 1 [file genes-14-01904-s001.zip › Figure S1.pptx]

## Slide 1
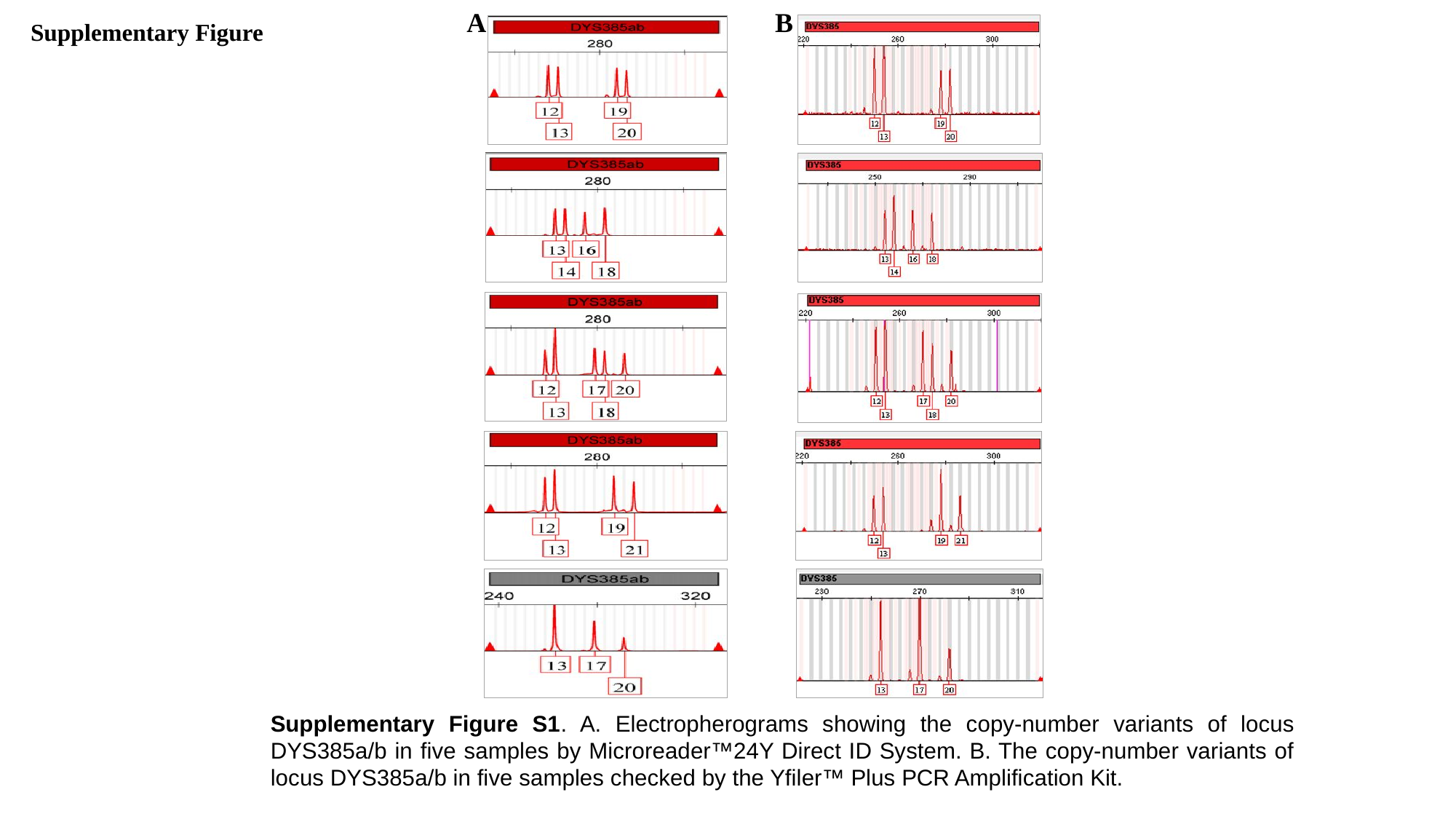

A
B
Supplementary Figure
Supplementary Figure S1. A. Electropherograms showing the copy-number variants of locus DYS385a/b in five samples by Microreader™24Y Direct ID System. B. The copy-number variants of locus DYS385a/b in five samples checked by the Yfiler™ Plus PCR Amplification Kit.
